# Medial Swivel Peritalar Fracture Dislocation: A Case Report

**DOI:** 10.7759/cureus.10529

**Published:** 2020-09-18

**Authors:** Ibrahim S ElMaghrby, Ahmed Mahmoud

**Affiliations:** 1 Orthopedic Surgery, Prince Mutaib Bin Abdulaziz Hospital, Al-Jouf, SAU; 2 Orthopedics and Traumatology, Ahmed Maher Teaching Hospital, Cairo, EGY; 3 Orthopedics and Trauma, King Salman Hospital, Riadah, SAU; 4 Orthopedics and Trauma, Al-Azhar University, Cairo, EGY

**Keywords:** equinovarus deformity, talonavicular dislocation, subtalar dislocation, calcaneocuboid dislocation, medial peritalar dislocation, acquired clubfoot, foot injury, midfoot injury, hindfoot injury

## Abstract

A medial swivel peritalar fracture-dislocation is a rare and disabling foot injury. The terminology describes a peritalar dislocation as the direction of peritalar foot displacement. Medial dislocation is the most frequent type. A rare variant involves talonavicular joint dislocation, subtalar joint fracture-dislocation, and calcaneocuboid fracture-dislocation. The clinical position of the foot resembles an equinovarus deformity. A computed tomography (CT) scan is necessary to obtain a diagnosis and formulate a surgical plan. A medial swivel peritalar fracture-dislocation is a challenging injury, and because there have been few reported cases in the literature, an optimal treatment protocol has not yet been established. We present a case of an unusual fracture-dislocation pattern of the hind and midfoot causing a complex talonavicular fracture-dislocation, subtalar fracture dislocation, and calcaneocuboid fracture-dislocation. The injury was successfully treated with open reduction and internal fixation.

## Introduction

Peritalar dislocation is the simultaneous dislocation of the distal articulations of the talus at the talonavicular and subtalar joints. The clinical literature on peritalar dislocation is limited. Peritalar fracture-dislocations are rare, disabling injuries, accounting for approximately 2% of all traumatic foot injuries [1].

Peritalar dislocation is defined according to the direction of the peritalar foot displacement. The medial and lateral swivel dislocations were the most common mechanism of injury. Main et al. introduced a classification for midtarsal injuries according to the direction of displacement and the type of injury [2]. Each of them presents their unique challenges to the treating surgeon.

Medial dislocation is the most common type, accounting for approximately 80% of cases reported in the literature [1]. It is usually caused by a high-energy mechanism such as a road traffic accident or a fall from a height. Injuries are often compound in nature or comprise one element of a poly-traumatized patient [3]. The clinical literature related to peritalar dislocation is limited, and the rarity of the occurrence of medial peritalar fracture-dislocations has prevented the establishment of a treatment protocol. The management is challenging, and the treatment is fraught with a high rate of disability, infection, and amputation [4]. A trial of a closed reduction under sedation is recommended. If closed reduction fails, surgery for open reduction and internal fixation may be required.

Herein, we present a case of a patient with a closed medial peritalar fracture-dislocation associated with osteochondral shearing injuries to the articular surface of the calcaneus and the navicular. The injury was successfully treated with open reduction and internal fixation, and the techniques used are thoroughly described.

## Case presentation

**Figure 1:**
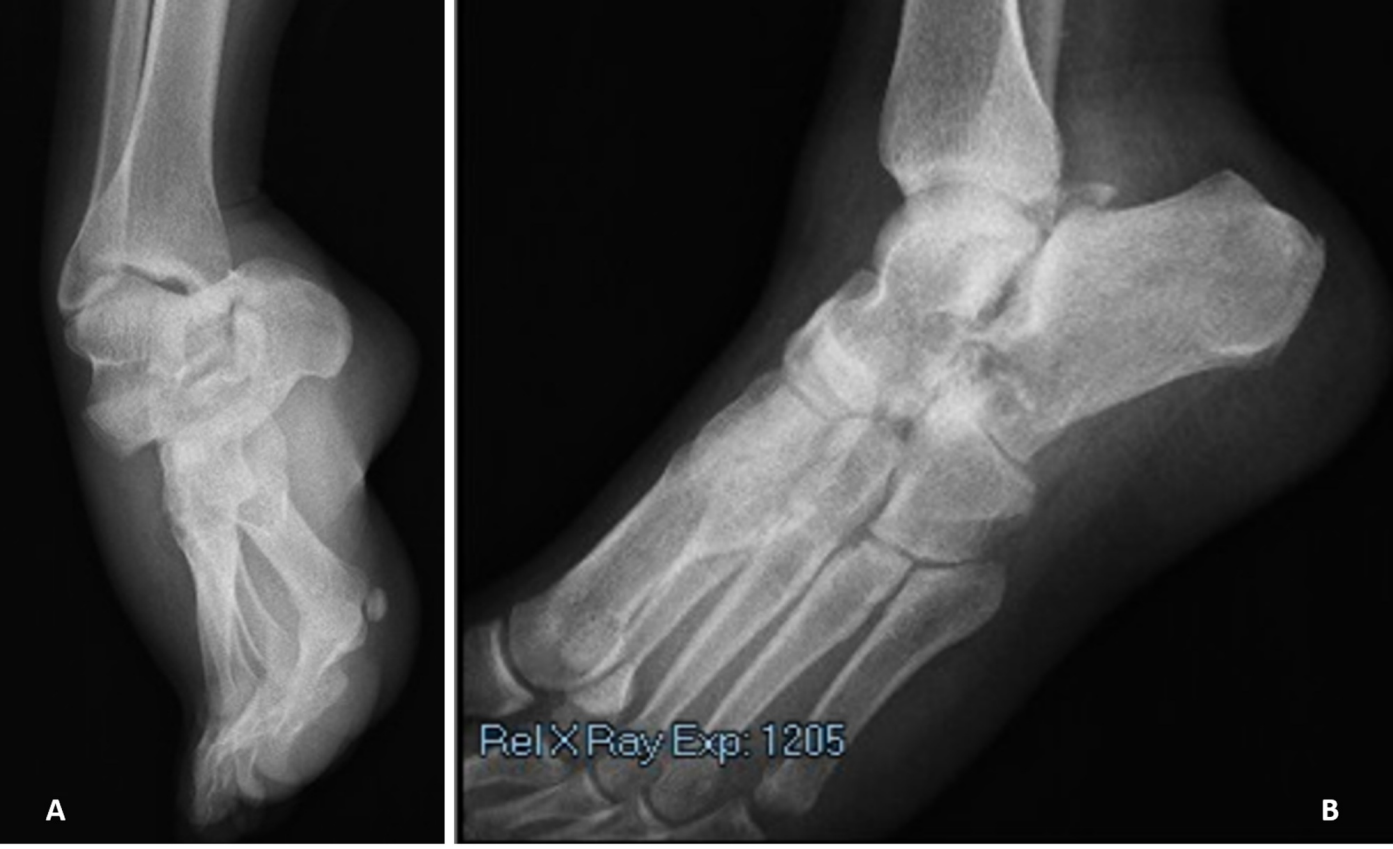
Preoperative (A) anteroposterior and (B) oblique radiographs of the injury. A large amount of equinovarus deformity, referred to as acquired clubfoot, is revealed.

**Figure 2:**
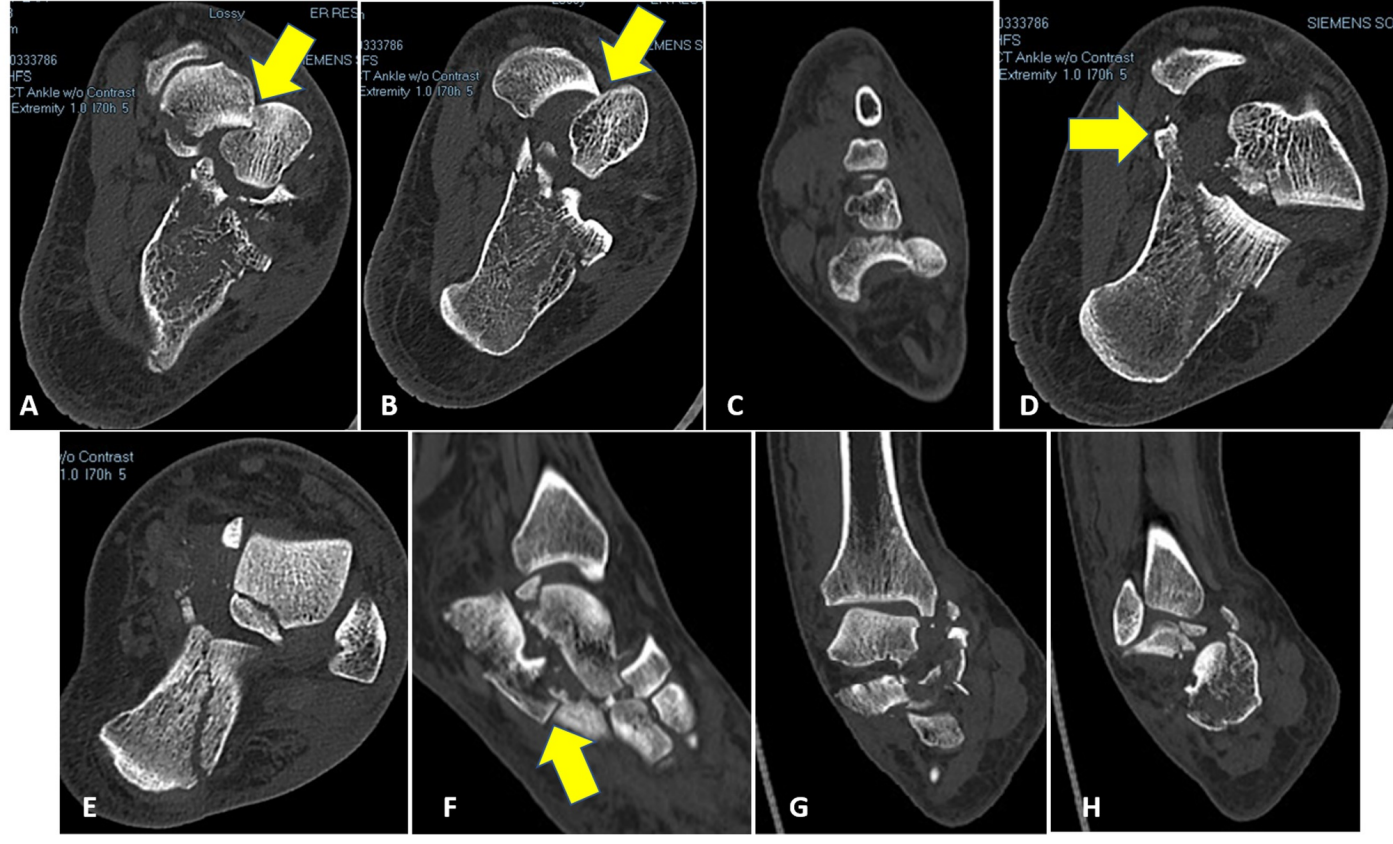
Preoperative (A-E) axial and (F-H) coronal computed tomography images showing radiographs of the injury (A, B) Yellow arrows showing the impaction of the lateral border of the navicular bone into the medial portion of the talar head. (D) Yellow arrow pointing to the fracture middle talar facet of the calcaneus. (F) Yellow arrow pointing to the calcaneocuboid fracture-dislocation.

**Figure 3:**
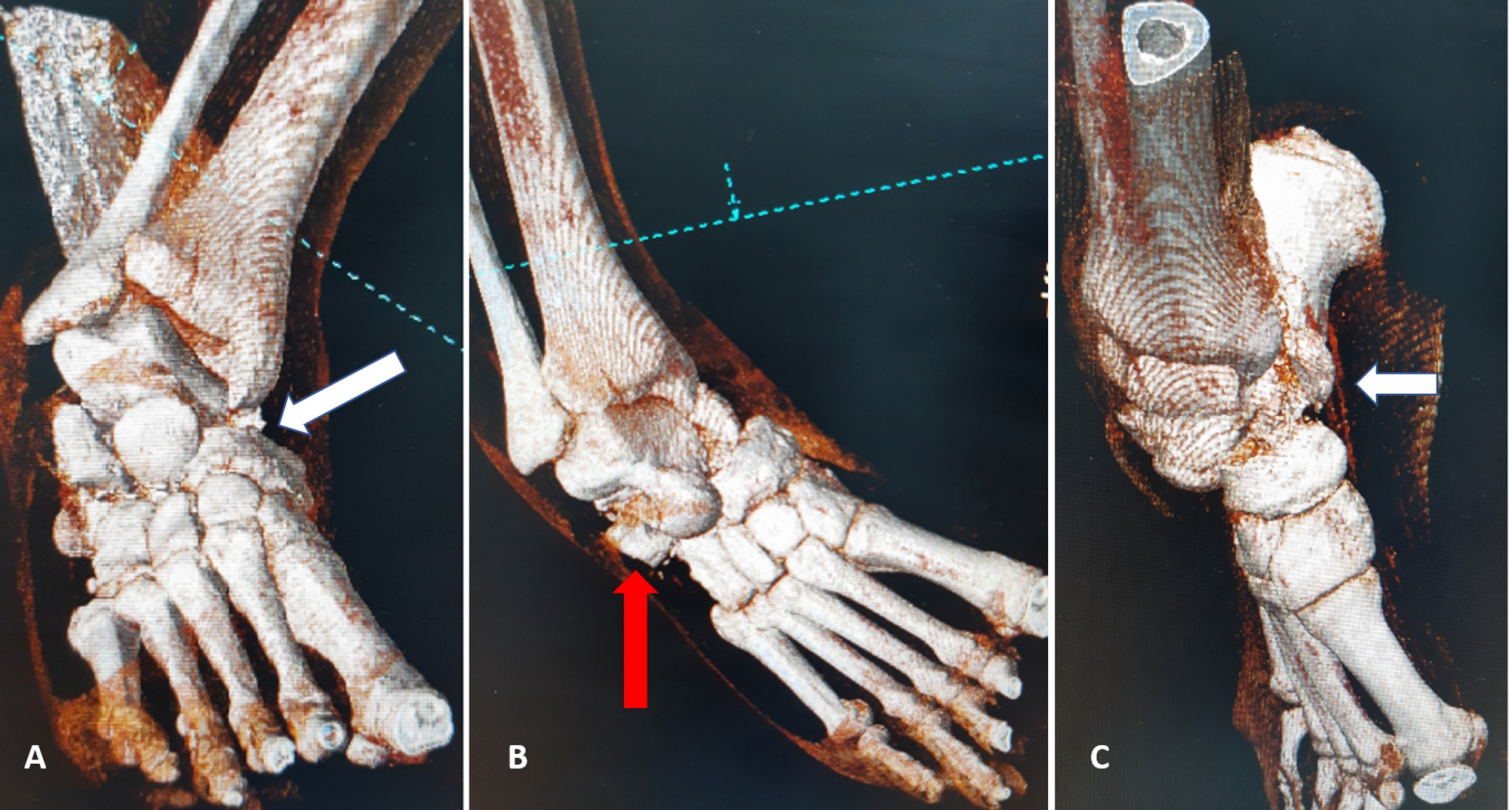
Preoperative three-dimensional computed tomography showing the total dislocation of the talonavicular joint (A, white arrow), an osteochondral fragment of the calcaneocuboid joint (B, red arrow), and the osteochondral fragment of the sustentaculum tali with the clinical appearance of acquired club foot injury (C, white arrow)

A 39-year-old man with an unremarkable medical and surgical history was brought to our emergency department by ambulance after a fall from a height. Our initial physical examination revealed a severe equinovarus deformity, which is referred to as acquired clubfoot. Swelling rapidly occurred and masked the bone deformity. Before the reduction of the dislocation, the man was evaluated for neurovascular impairment. He was unable to bear weight. Initial extremity radiographic films are shown in Figures 1A-1B, and computed tomography (CT) scans were obtained as shown in Figures 2A-2E and Figures 3A-3C.

The patient was diagnosed with a medial peritalar dislocation with multiple complex injuries, including a closed talonavicular fracture-dislocation. There was a small fracture of the medial part of the talar posterior facet, an undisplaced talar neck fracture, a calcaneocuboid fracture-dislocation (Figure 2F), and a subtalar joint fracture-dislocation with a fracture of the middle talar facet of the calcaneus (sustentaculum tali; Figure 2D), with intact distal vascularity of the right foot and soft compartments. In addition to the foot injury, there was a fracture-dislocation of the right shoulder and undisplaced fracture of the right head radius.

**Figure 4:**
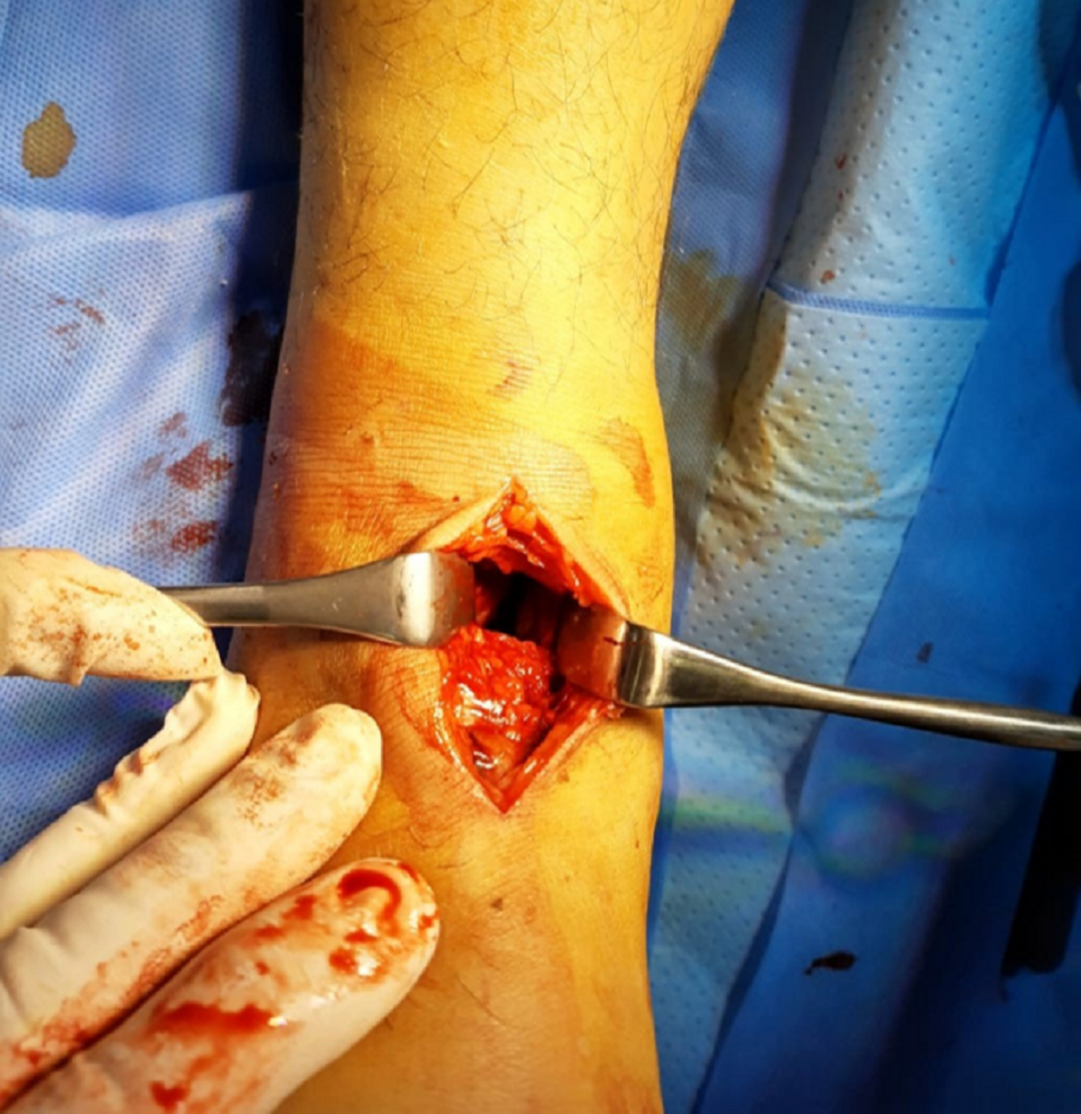
Surgical photograph showing the anteromedial approach over dislocated talonavicular joint

We immediately performed surgery on the patient for dislocation reduction. With the patient under general anesthesia, a dose of prophylactic antibiotics was given. After prepping and draping, an immediate open reduction was undertaken to correct the dislocation through an anteromedial approach over the talonavicular joint. The extensor halluces longus tendon and dorsalis pedis artery were laterally retracted to reach the talonavicular joint (Figure 4).

A single Schanz pin was inserted from the medial to the lateral direction into the navicular for the disimpaction of the lateral navicular wall and talar head (the traction and counter traction technique; Figure 2A). Reduction was achieved when the navicular bone was externally rotated and adducted to disimpact the lateral edge of the lateral navicular wall, and then the abduction of the forefoot was performed. A post-reduction examination under fluoroscopic vision was performed. The talonavicular joint was stabilized with two 1.6-mm Kirschner (K) wires placed from the navicular bone to the talus. The talar neck fracture was fixed by a 4-mm cannulated partially threaded screw and a 1.6-mm K-wire.

**Figure 5:**
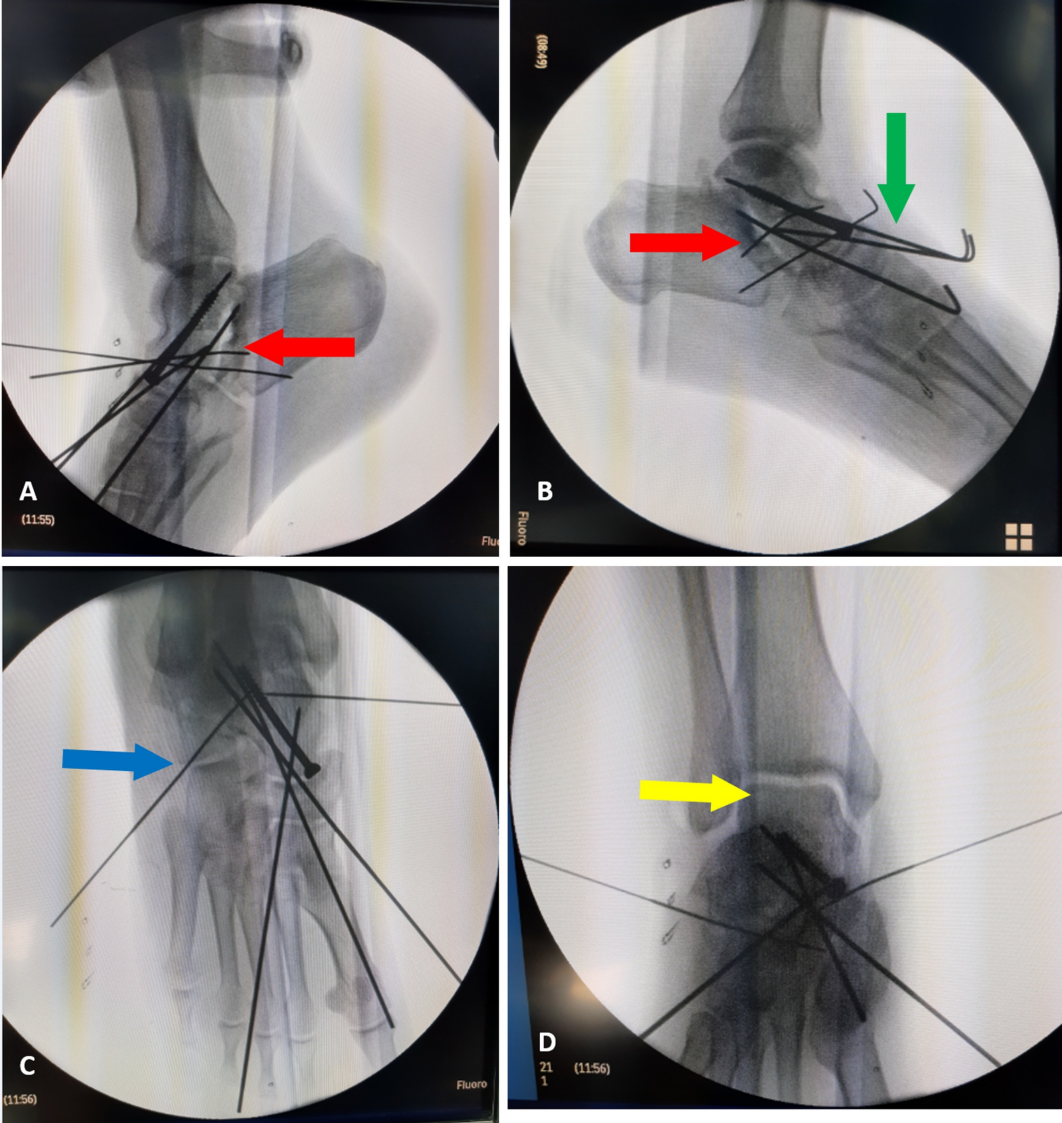
Immediate postoperative lateral radiograph (A, B) of the foot and ankle showing anatomical reduction of the talonavicular joint (green arrow) and subtalar joint (red arrow). An anteroposterior radiograph (C, D) of the foot and ankle showing the anatomical reduction of the calcaneocuboid joint (blue arrow) and the restoration of the ankle mortise (yellow arrow)

The osteochondral shearing fragment of the calcaneal middle talar articular surface (sustentaculum tali) and the fragment of the cuboid facet of the calcaneus were reduced anatomically by the joystick technique, fixed by a 1.4-mm K wire. The wound was closed by the Allgower-Donati suture technique. A short leg plaster was applied. The patient was instructed to perform non-weight bearing exercises using the right leg for eight weeks. Postoperative radiographs revealed an anatomical reduction of complex injuries (Figures 5A-5D)

Clinically, the patient remained well, with no complications except for small raw areas of skin at the location of incisions.

**Figure 6:**
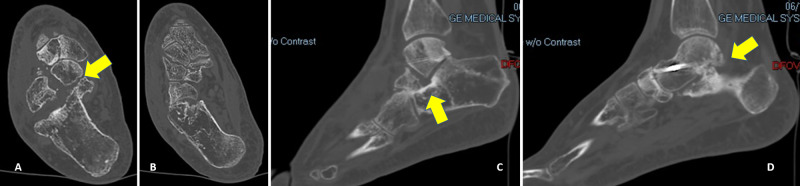
Postoperative computed tomography images (A-D) at eight months showing a stable talonavicular joint, a united calcaneus fracture, and osteoarthritis at the posteromedial aspect of the subtalar joint (yellow arrows)

**Figure 7:**
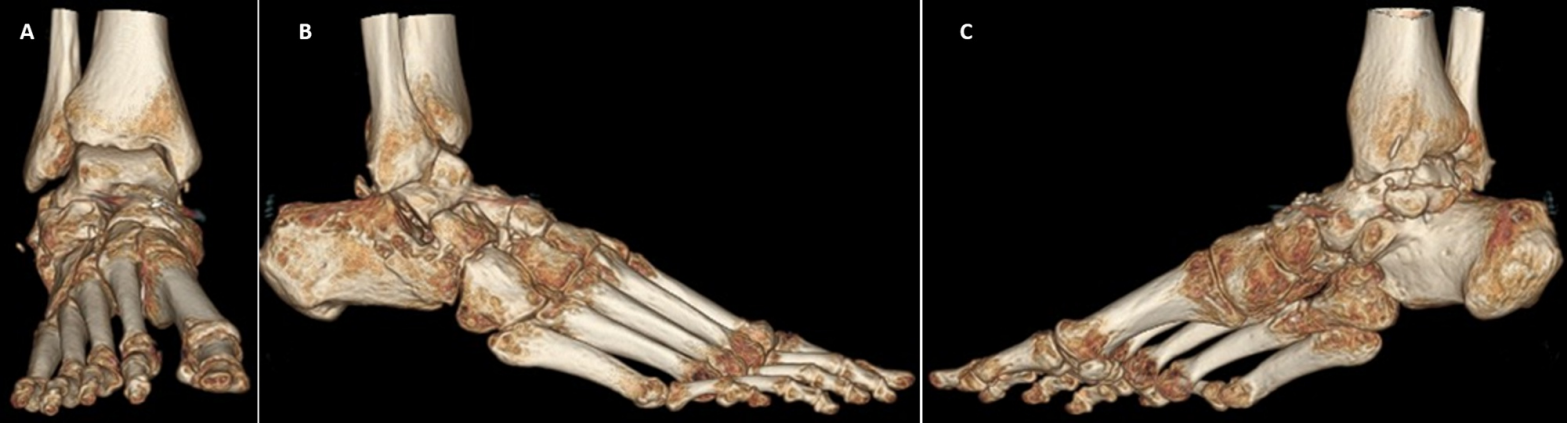
Three-dimensional computed tomography at eight months showing anatomical reduction of the talonavicular, calcaneocuboid, and subtalar joints with mild osteoarthritic changes of the posteromedial side of subtalar joint (A-C)

The K-wires and plaster were removed eight weeks after surgery. The patient was allowed to bear weight, with a gradual increase in load. At his last follow-up, after eight months, he had successfully returned to normal activities. His foot was stable, and he reported concerns of only mild pain on the posteromedial side of his hind foot after long walks. The subtalar joint movement was mildly limited, and the talonavicular joint was considered normal. Osteoarthritis appeared at the posteromedial aspect of the subtalar joint on radiographs, and CT scans were obtained eight months postoperatively (Figure 6A-6D, Figure 7A-7C).

## Discussion

A medial peritalar fracture-dislocation is not a common injury. It is a potentially limb-threatening injury with a very poor prognosis. The hallmarks of these injuries were described by Ricci et al. [4], and controversy remains regarding the most appropriate management [5]. To the best of our knowledge, the largest published series to date includes 15 feet [6-8]. A medial peritalar dislocation presented in nearly four of every five of the cases reported in the literature [1].

These injuries are usually caused by high-energy trauma such as a fall from a height or a motor vehicle injury [2]. Main et al. introduced a classification for midtarsal injuries according to the direction of displacement and the type of injury [2]. In their study, all displacement types were discussed, including medial, lateral, longitudinal, plantar, and crush injuries. Medial swivel dislocations have also been incorporated in the category of medial displacements [9]. Medial peritalar dislocations are associated with fractures of the hind, mid, and forefoot [10]. Osteochondral shearing injuries to the articular surface of the talus, the calcaneus, or the navicular are common [11]. These injuries are difficult to detect on plain radiographs [12].

In medial swivel peritalar dislocations, an inversion force applies to a plantarflexed foot. The sequence of the injuries starts with talonavicular joint dislocation followed by the dislocation of the subtalar joint. Then, a calcaneocuboid fracture-dislocation appears. The final clinical appearance of the foot mimics an acquired clubfoot deformity. The position of the head of the talus is immobilized between the extensor hallucis longus and the long-toe extensors [1].

We present the unique finding of a case of hind and midfoot injury with a unique configuration of a medial swivel fracture-dislocation after a fall from a significant height. The accurate diagnosis and understanding of these injuries are difficult when using plain X-rays due to the superposition of the hindfoot and midfoot bones. A CT scan is recommended as part of the diagnostic protocol without delaying initial emergency management. The CT scan assists with delineating subtle fractures and dislocations, and it is also necessary for devising a surgical plan. Associated injuries are present in almost all cases. Fortunately, in this case, there was no neurovascular deficit. Treatment constructs for a medial peritalar dislocation have included immediate open reduction and internal fixation with K-wires. With closed reduction, internal fixation is performed for the osteochondral calcaneal fragment of the calcaneocuboid joint, the sustentaculum tali, and the fractured neck talus. Our patient exhibited multiple negative prognostic features, including subtalar and calcaneocuboid incongruity, and disruption of heel width, height, and alignment [6-8,13].

Studies have reported various options for treating the dislocation of the talonavicular joint. These options are either open or closed reduction with or without internal or external fixation. Open reduction and internal fixation are good options if a failed attempt of closed reduction and the talonavicular joint is unstable after reduction. Richter et al. found that the group with closed reduction without internal fixation has similar outcomes to other methods [14]. Moreover, they recommended open reduction as an initial strategy given its favorable outcomes compared to closed reduction [15].

Most studies recommended that joint stabilization be performed by internal fixation for a dislocated Chopart joint due to the risk of vascular injury. Providers must be familiar with the recognition and treatment of these injuries to limit damage to the articular surfaces of the foot and ankle from improper diagnosis or failed attempts at reduction [6-8,13]. Prompt recognition and rapid reduction of fractures and dislocations provide the best possible outcome. Delay in treatment will cause an increased risk of soft tissue complications, avascular necrosis, infection, amputation, osteoarthritis, and possible equinovarus deformity development in missed and untreated cases [16]. A closed reduction can be performed under intravenous sedation in the emergency room. Concomitant comminuted calcaneal fractures generally have poor outcomes following either conservative or operative treatment and, thus, nonoperative management seemed prudent [16].

In such injuries, subtalar osteoarthritis is likely to develop, leading to restriction of movements. Early mobilization and an intensive rehabilitation protocol are needed to obtain an excellent functional outcome. In this case, compartment syndrome and loss of soft tissue coverage were not major problems.

In our experience, the role of closed reduction of medial peritalar cases involving talonavicular dislocation and regional fractures is limited in cases where there is an impaction of the lateral edge of the navicular into the medial wall of the talar head (Figure 2A). Closed reduction without proper disimpaction may lead to an iatrogenic fracture of the neck of the talus.

## Conclusions

This case represents a rare injury and its effective treatment. Our treatment approach began with an attempt at a closed reduction for a medial swivel dislocation of the talonavicular joint. In the event of closed reduction failure, we implement the open reduction method to avoid an iatrogenic talar neck fracture. In the presence of a fracture of the middle talar facet of the calcaneus and calcaneocuboid fracture-dislocation, we recommend the use of anatomic reduction and internal fixation to restore joint congruity and reduce the risk of osteoarthritis.
